# *Cassia angustifolia* Vahl. Leaves: Determination of Total Phenolic and Sennoside Contents of Different Fractions in Comparison with Their α- Glucosidase and Tyrosinase Inhibitory Effects

**DOI:** 10.5812/ijpr-140914

**Published:** 2024-03-09

**Authors:** Dina Morid Ahmadi, Somayeh Mojtabavi, Shima Ghadami, Mahdieh Eftekhari, Mohammad Reza Shams Ardekani, Mohammad Ali Faramarzi, Mahnaz Khanavi

**Affiliations:** 1 Department of Pharmacognosy, Faculty of Pharmacy and Persian Medicine and Pharmacy Research Center, Tehran University of Medical Sciences, Tehran, Iran; 2 Department of Pharmaceutical Biotechnology, Faculty of Pharmacy, Tehran University of Medical Sciences, Tehran, Iran; 3 Pharmaceutical Sciences Research Center, Kermanshah University of Medical Sciences, Kermanshah, Iran; 4 Department of Pharmaceutical Biotechnology, Biotechnology Research Center, Faculty of Pharmacy, Tehran University of Medical Sciences, Iran

**Keywords:** *Cassia angustifolia* Vahl., Tyrosinase, α-Glucosidase, Total Phenol, Sennoside B

## Abstract

**Background:**

*Cassia angustifolia* Vahl. (*Senna*) is a medicinal plant containing anthraquinone compounds such as sennoside. *Senna* is primarily valued for its laxative properties. In Persian medicine, this plant has been also used to treat various disorders such as diabetes and skin hyperpigmentation. Previous studies have shown that different species of *senna*, such as C. articulata, C. alata, *C. Siamea*, *C. Surattensis* inhibit alpha-amylase and α-glucosidase enzymes. To the best of our knowledge, no previous evidence is available on tyrosinase and α-glucosidase inhibitory effects of the extract and different fractions of *C. angustifolia* leaves.

**Objectives:**

The purpose of this study was to investigate the inhibitory effect of the methanol-water extract and different fractions (hexane, chloroform, ethyl acetate, and remaining crude extract) of *senna* against tyrosinase and α-glucosidase and to investigate their total phenolic and sennoside B contents.

**Results:**

Our findings depicted that the methanol-water extract and fractions had no significant anti-tyrosinase activity; however, some fractions were active toward α-glucosidase. The hexane fraction and the remaining crude extract demonstrated the highest inhibition on α-glucosidase compared to acarbose (positive control). In addition, the ethyl acetate fraction contains high phenolic and hydroxy anthraquinone derivatives based on the amount of sennoside B contents equivalent to 382.25 μg/mL of gallic acid and 1.525% of sennoside B, respectively. Moreover, no correlation was observed between the phenolic and sennoside contents of different fractions and their α-glucosidase inhibitory effect.

**Conclusions:**

Considering the α-glucosidase inhibition results, the hexane fraction of *C. angustifolia* can be a valuable fraction for in vitro and in vivo antidiabetic studies as well as further phytochemical studies. Further studies to identify the active substances and the exact mechanism of the bioactive ingredients on the inhibitory effects of α-glucosidase can provide promising results in the future.

## 1. Background

*Cassia L.*, the largest genus in the *Caesalpinioideae* subfamily, has nearly 600 flowering species worldwide. *Cassia angustifolia* Vahl. (*C. angustifolia*), or *Senna* is a small medicinal shrub that is valuable for its drought resistance. *Senna* is grown as a cash crop worldwide. Its medicinal uses were first described in the writings of 9th-century Arabian physicians Serapion and Mesue, and the name *Senna* is of Arabic origin. Similarly, *Senna*, as a medicinal herb, has been utilized in Greek, Ayurveda, homeopathy, and allopathy medicine. It is also used as a febrifuge in splenomegaly, anemia, typhoid, cholera, biliousness, jaundice, gout, tumors, bronchitis, and leprosy (1).

*Senna* is an aromatic shrub native to Egypt, Sudan, Nigeria, and North Africa, as well as India, Pakistan, and China. The volatile oil of *C. angustifolia* leaves contains aldehydes, terpenes, and phenols. Sodium potassium tartrate, essential oil, mannitol, saponin, and resin are also present in the plant leaves (2). Additionally, it contains Isorhamnetin and kaempferol. Its leaves and pods contain anthraquinone sennosides and glycosides (3). Sennosides A and B, as optical isomers, have been isolated from the leaves and pods of this plant. Besides these sennosides, the presence of sennosides C, D, and G is reported and extensively used as laxatives (4). Three sennidins are also present in the leaves. The leaves, pods, and roots contain rhein, chrysophanol, emodin, and aloe-emodin. Several mono and di-glucosides of anthrone exist in the seedlings, leaves, and roots. The leaves further contain the mono-β-D glucosides of rhein, aloe-emodin, and water-soluble components, which are supposed to be responsible for the synergistic effects (5).

Alpha-glucosidase is a vital enzyme found in the human body. Inhibitors of alpha-glucosidase play a crucial role in managing postprandial hyperglycemia (PPHG) in diabetic patients because inhibiting the activity of the alpha-glucosidase enzyme decreases starch hydrolysis (6). The efficacy, safety, cardiovascular benefits, and absence of hypoglycemia make alpha-glucosidase inhibitors (AGIs) suitable as first-line therapy in diabetes (7). Consequently, the mechanism of alpha-glucosidase inhibition delays the absorption of carbohydrates, leading to a reduction in protein glycation and, consequently, lower levels of hemoglobin and glycated end-products in collagen. This process improves biochemical parameters and provides protection against diabetic neuropathy and nephropathy (8). Compounds with alpha-glucosidase inhibitory (AGI) properties, such as saponins, glycosides, terpenoids, and flavonoids, can be found in species such as *C. articulata*, *C. alata*, *C. Siamea*, *C. Surattensis*, and *C. semen*.

An ethanol extract of *C. articulata* at a dose of 0.45 g/kg demonstrated significant antihyperglycemic effects, regulating serum glucose (113.3 ± 10.30 mg/dL), plasma insulin (14.16 ± 0.67 μU/mL), total hemoglobin (11.5 ± 0.91 g/dL), and glycosylated hemoglobin (0.37 ± 0.04 mg/gHb), compared to streptozotocin-diabetic rats and the standard antidiabetic glibenclamide. Another study investigated the methanol extract of *C. alata* and its fractions (ethyl acetate and butanol fractions), revealing significant α-glucosidase inhibitory activity (IC50 levels = 63.75 ± 12.81, 2.95 ± 0.47, and 25.80 ± 2.01 μg/mL, respectively), which were higher than the reference drug acarbose (IC50 level = 107.31 ± 12.31 μg/mL). Two flavonoids isolated from the ethyl acetate and butanol fractions of *C. alata*, kaempferol (IC50 level = 56.7 ± 7.7 μM) and kaempferol 3-O-gentiobioside (IC50 level = 50.0 ± 8.5 μM), exhibited significant α-glucosidase inhibitory activity compared to acarbose (8, 9).

Despite some investigations into the properties of various extracts of *C. angustifolia* to inhibit the α-glucosidase enzyme, no research has examined different fractions obtained from *C. angustifolia* leaves to date. Likewise, there are no reports existing regarding the measurement of the inhibitory power of the tyrosinase enzyme by the extract and fractions of *C. angustifolia* leaves.

## 2. Objectives

We investigated the inhibitory effects of the extract and its fractionations on inhibiting tyrosinase and α-glucosidase enzymes.

## 3. Methods

### 3.1. Chemicals

α-Glucosidase, p-nitrophenyl α-D-glucopyranoside (P-NPG), tyrosinase, kojic acid, gallic acid, acarbose, and catechol were obtained from Sigma-Aldrich, Germany. Methanol, hexane, ethyl acetate, chloroform, dimethyl sulfoxide (DMSO), K_2_HPO_4_.3H_2_O, KH_2_PO_4_, and potassium hydroxide were purchased from Merck®, Germany.

### 3.2. Plant Material

The dried leaves of *C. angustifolia* were purchased from an Iranian medicinal plant market in February 2018. The plant material was botanically authenticated by botanists (Popular Market Plant, PMP-453) in the herbarium of the Faculty of Pharmacy, Tehran University of Medical Science. Subsequently, the leaves were powdered using a chopper (Toshkan, Iran).

### 3.3. Plant Extract Preparation

**Figure 1.**
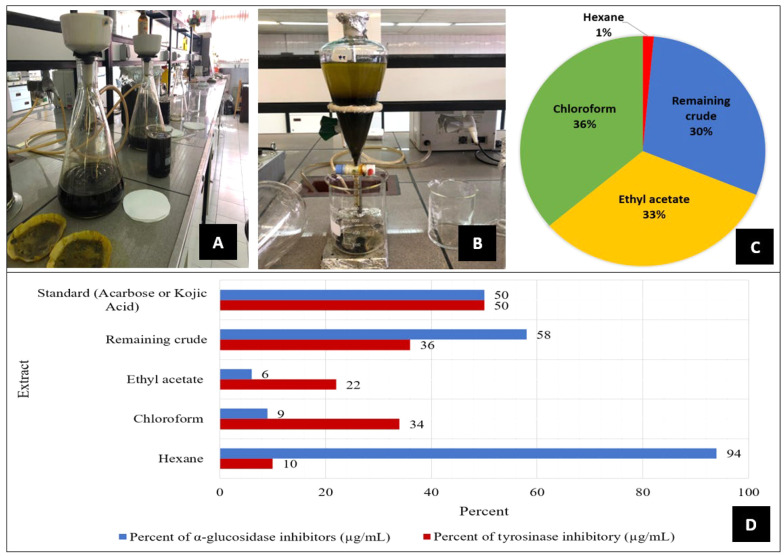
A, filtration of the methanol-water extract of *Senna*; B, fractionation of the *Senna* extract; C, percentage composition of components in the whole *Senna* extract; D: Comparison of inhibition percentages of the remaining crude extract and fractions of *Senna* leaves (500 μg/mL), and standard (484 μg/mL) in α-glucosidase, and standard (500 µg/mL) tyrosinase enzyme inhibition.

A total of 500 grams of the powdered plant leaves were extracted with 6000 mL methanol/water extract 80:20 (MWE) using the maceration method for 3 × 72 hours at room temperature in three steps (Figure 1A). Furthermore, the solvent was removed using rotary evaporation (Heidolph, Germany), followed by drying the residue solvent with a freeze dryer (Christ, Germany). Moreover, the MWE was fractionated by hexane, chloroform, and ethyl acetate, respectively (Figure 1B), which included hexane, chloroform, and ethyl acetate fractions and the remaining crude extract.

### 3.4. Glucosidase Inhibitory Activity

The α-glucosidase inhibitory activity of the fractions and MWE was assessed as previously described with minor modifications (10). The reaction mixture, including 20 μL of α-glucosidase (1 U/mL), 135 μL of phosphate buffer (50 mM, pH 6.8), and 20 μL of different concentrations of the fractions and MWE (0.1 - 0.5 mg/mL), was preincubated in a 96-well plate at 37°C for 10 minutes. After adding 25 μL of P-NPG (4 mM) as a substrate (11), incubation was carried out at 37°C for 20 minutes. The absorbance of released p-nitrophenol was determined at 405 nm using a microplate reader. Acarbose was used as the standard at different concentrations (0.1 - 0.5 mg/mL). The experiments were conducted in triplicate, and the results were reported as percentage inhibition calculated using the below formula:


$$Inhibitory\;activity\;(\%)\;=(1-\frac{A_{s}}{A_{c}})\times 100$$

A_s_ indicates the absorbance in the presence of the test substance, and A_c_ indicates the absorbance in the control.

### 3.5. Tyrosinase Inhibition Assay

This assay was conducted based on the procedure by Uchida et al. with slight modifications. Initially, a 10 µL solution of various concentrations of the MWE and fractions, prepared in DMSO, was added to a 96-well microplate, followed by mixing with 180 µL of phosphate buffer (1.15 M, pH 6.8) on ice. Then, 100 µL of l-DOPA (100 µg/mL) in phosphate buffer and 100 µL of mushroom tyrosinase (1000 U/mL in phosphate buffer) were added. The mixture was incubated at 37 °C for 20 minutes, and the level of dopachrome generation was assessed spectrophotometrically at 492 nm using a microplate reader. Kojic acid was used as the positive control, and the concentration for 50% inhibition (IC50) was calculated. All measurements were conducted at least three times (12).

### 3.6. Assessment of Total Phenolic Content

The total phenolic content of the extract and fractions of *C. angustifolia* were measured using standard methods involving the gallic acid standard and Folin-Ciocalteu’s phenol reagent (13). A solution (0.1 mL) containing the extract or fractions (1000 µg) was pipetted into a 50 mL volumetric flask, followed by the addition of 1 mL of Folin-Ciocalteu’s phenol reagent and 46 mL of distilled water (DW). The flask was then shaken.

After 3 minutes, 3 mL of the 2% Na_2_CO_3_ solution was added, and the mixture was left for 2 hours with periodic shaking. Absorbance measurement was then conducted at 760 nm. A similar experiment was performed for all standard gallic acid solutions (0 - 1000 mg/0.1 mL), and a standard curve was constructed using the equation below (14).


$$Absorbance=0.0012\;gallic\;acid\;\left(\mu g\right)+0.0033\;(1.25)\;$$

Additionally, the total phenol content of the extract or fractions, expressed as gallic acid equivalent, was determined using the absorbance value measured at 760 nm and inputting it into the equation derived from the standard curve. The experiment was conducted in triplicate, and the equivalent value of gallic acid was reported as the mean ± SD of the triplicates (15).

### 3.7. Total Anthraquinone Glycosides Analysis

Rhein, as the major component, was used as a standard for quantitative total anthraquinone glycosides analysis (3). A calibration curve for the Rhein standard was prepared at 5 concentrations (2.00 × 10^-6^ to 10.00 × 10^-6 ^g/mL). Additionally, 0.5% w/v of magnesium acetate was added, and the volumes were adjusted with methanol. These concentrations were measured using the UV-VIS spectrophotometric method at 515 nm (Cintra 6, GBC, Australia), and the relationship between concentration and absorbance was plotted accordingly. Linearity was assessed by regression analysis, residual sum of squares, and correlation coefficient (r^2^) calculation. The procedure for the analysis of total anthraquinone glycosides was based on the method for hydroxy anthracene derivatives of *C. angustifolia* as described in the Standard of ASEAN Herbal Medicine (ASEAN Countries, 1993).

### 3.8. Statistical Analysis 

All assessments were performed three times and expressed as the mean ± standard deviation (SD). Inhibitory concentration (IC50) values were calculated using Microsoft Excel statistical software, version 2013.

## 4. Results

According to the results, 80% of the methanol-water extract (MWE) of this plant was obtained with a yield of 24.8%. The remaining crude extract (26.78 g) was obtained during the fractionation process of the MWE using hexane (1.31 g), chloroform (32.61 g), and ethyl acetate (29.30 g) solvents, respectively.

Investigating different fractions of *C. angustifolia* using the in-vitro α-glucosidase inhibitor test with a concentration of 484 µg/mL, the hexane fraction and the remaining crude extract exhibited the highest inhibition at 52 and 58 µg/mL, respectively, which was more effective than the inhibition caused by acarbose. The concentration of acarbose used was 750 µM (equivalent to 484 µg/mL). Although the concentration of acarbose applied in this measurement was lower than that of the extract, the observed results unexpectedly reached nearly 50%.

The present study evaluated the fractions of the MWE of *C. angustifolia* for their inhibitory effects on tyrosinase and α-glucosidase enzymes. The results showed no significant inhibitory effect on the tyrosinase enzyme (Table 1). 

**Table 1.** Results of Tyrosinase Inhibitory, α-Glucosidase Inhibitory, Total Phenolic, Total Anthraquinone Glycosides of *Cassia angustifolia* Fractions

Extract	Percent of Tyrosinase Inhibitory Effect (500 µg/mL)	Percent of α-Glucosidase Inhibitors (484 µg/mL)	Total Phenolic Content (µg/mL)	Percent of Total Anthraquinone Glycosides (%)	
				Drug ^a^	Extract
**Hexane fraction**	10	94	372.36	0.577	0.447
**Chloroform fraction**	34	9	368.64	0.425	0.318
**Ethyl acetate fraction**	22	6	404.88	1.379	1.127
**Remaining crude extract**	36	58	374.04	0.438	0.688
**Acarbose (750 µM)**	-	50	-	-	-
**Kojic acid (82 µM)**	50	-	-	-	-

^a^ C-Lax herbal tablet.

The results demonstrated that the fractions of MWE had no significant inhibitory effect on the tyrosinase enzyme, but hexane fraction and the remaining crude extract can be selected as valuable fractions for further studies to find active compounds as α-glucosidase enzyme inhibitors.

Based on absorbance values obtained from the reaction of the fractions of MWE with the Folin-Ciocalteu reagent and their comparison with the absorbance values of gallic acid as a standard, the total amount of phenolic compounds was estimated to be approximately 382.25 µg/mg for the ethyl acetate fraction, and 290.58 µg/mg and 288.08 µg/mg for the remaining crude extract and chloroformic fraction, respectively.

We determined the percentage of sennoside B in the extract according to the ASEAN-herbal-medicine standard (11); the highest percentage was observed in the ethyl acetate fraction, indicating that ethyl acetate was more effective in extracting phenolic compounds compared to the other fractions.

## 5. Discussion

*C. angustifolia* flowers exhibited an ability to inhibit the tyrosinase enzyme, whereas the extracts of the leaves failed to inhibit this enzyme in the present study. According to the results of the α-glucosidase inhibitory assay, the hexane fraction and the remaining crude extract can be valuable fractions for further studies to identify active compounds as α-glucosidase enzyme inhibitors (Figure 1D). Notably, no phytochemical analysis has been conducted on the hexane fraction of this plant to date. Alloxan increased the levels of total cholesterol, urine ketones, FBG, VLDL-cholesterol, LDL-cholesterol, and cardiac function indices while reducing the levels of globulin, HDL-cholesterol, albumin, hexokinase, liver glycogen, and glucose-6-phosphate dehydrogenase activities. The ethyl acetate fraction reversed the levels and/or activities of these biochemical indices to those of non-diabetic animals treated with DW. Emodin (1, 3, 8-trihydroxy-6-methylanthraquinone), a bioactive constituent available in the* C. alata* flower, produced the highest α-glucosidase inhibitory effects (16).

The *C. singueana* extract exhibited appropriate values for phenolic content (120.01 mg GAE/g dry extract), DPPH (IC50: 9.35 μg/mL), and α-glucosidase inhibition (I%: 90.47%) (17). In a phytochemical screening performed on the methanolic extract of *C. angustifolia* root and its fractions, the hexane fraction showed qualitative amounts of terpenoids, anthraquinones, and coumarins (18). However, it is unlikely that coumarins in this plant represent antidiabetic activity. Another plant of this genus, *C. alata* crude extract, suppressed α-glucosidase (IC50: 63.75 ± 2.81 μg/mL), whereas kaempferol-3-O-gentiobioside had an IC50 of 82.5 ± 13.7 μg/mL. Moreover, the *C. articulata* ethanolic seed extract suppressed yeast α-glucosidase (IC50: 149.6 ± 0.21 μg/mL) (8).

Evidently, the ethanol extract of *C. auriculata* flowers administered at 0.45 g/kg for 30 days to male Wistar rats exhibited a significant antihyperglycemic effect, normalizing plasma insulin, serum glucose, glycosylated hemoglobin, and total hemoglobin levels to 14.16 ± 0.67 μU/mL, 113.3 ± 10.30 mg/dL, 0.37 ± 0.04 mg/gHb, and 11.5 ± 0.91 g/dL, respectively (19). *C. angustifolia*, a natural laxative medicinal plant, contains sennosides (anthraquinone glycosides) as natural products (20). Variations in the total sennoside content (%) with the ontogeny of the leaves indicate that sennosides are abundant in the youngest leaves. The decrease in sennoside content from the youngest leaf to the leaf at the seventh node suggests the young leaf is a crucial tissue for assessing sennoside transport and biosynthesis (20). The presence of sennoside B: 1.55%, sennoside A: 0.73%, and rutin: 0.15% in *C. angustifolia* Vahl was indicated by HPLC method (21). *C. fistula* Linn contains sennosides A and B (11).

The combined treatment with aqueous leaf extract of *C. angustifolia* (EACA) and seed extract of *Foeniculum vulgare* (EAFV) in decreasing the severity of diabetes mellitus and oxidative stress in streptozotocin-induced diabetic male rats was tested. Each EACA and/or EAFV could be used as an antidiabetic complement in cases of DM due to their antioxidative properties (22). According to a phytochemical investigation, flavonoids, glycosides, and saponins are present in the leaf extracts of *C. angustifolia* and *Raphanus sativus* Linn. Gravimetric analysis for both extracts indicated the presence of flavonoids: 4.62%, glycoside: 2.62%, and saponins: < 2% in the *C. angustifolia* extract. These extracts ameliorate diabetic metabolic abnormalities and reduce the risk of complications due to chronic hyperglycemia. Thus, treatment with these two extracts caused a significant improvement in dyslipidemia due to the presence of various phytoconstituents and enzyme inhibitory actions, and the treatment was comparable to metformin treatment (21).

*C. fistula* Linn is utilized for treating pruritus, hematemesis, leukoderma, intestinal disorders, and diabetes and as a laxative, antipyretic, and analgesic in folk medicine (23). It contains high levels of phenolic antioxidants such as flavonoids, anthraquinones, and flavan-3-ol derivatives. Additionally, it contains chrysophanic acid, emodin, phenolic, ferulic acid, proanthocyanidin, rheinglucoside, rhein, galactomannan, phlobaphenes, tannin, oxy anthraquinone substances, fistuacacidin, lupeol, barbaloin, hexacosanol, and beta-sitosterol (23). The aqueous extract of *C. fistula *seeds, flowers, stem bark, and whole parts of *C. fistula* have shown antidiabetic activity in alloxan-induced diabetic rats by controlling sugar levels. Methanolic and aqueous extracts of this plant in normoglycemic and streptozotocin-nicotinamide-induced Type 2 diabetic rats demonstrated hypoglycemic activity (23).

Different parts of *C. auriculata* are used to treat various ailments, and reportedly, it possesses antimicrobial, antipyretic, anthelmintic, hepatoprotective, and antihyperlipidemic activities (19). Its phytoconstituents, such as flavonoids, alkaloids, polysaccharides, anthracene derivatives, and tannins, have pharmacological effects for treating diabetes, asthma, ulcers, conjunctivitis, liver and kidney disorders, cancer, and skin problems (24).

*C. auriculata* is of high nutritional value and offers health benefits. Reportedly, the methanolic extract of *C. auriculata* root had the highest total flavonoid and polyphenol contents and exhibited the highest antidiabetic and antioxidant effects in vitro compared to other extracts, which can be attributed to the presence of coumaric acid and -OH groups. Nonetheless, the ethanolic extract of *C. auriculata* root (administered at 150 mg/kg body weight) normalized the condition of mice with type 2 diabetes similar to the control mice. Hence, the extract is applicable as a strong antidiabetic and antioxidant agent in the pharmaceutical industry (25). Cassia plants are rich sources of anthraquinones, which are anthracene derivatives with a parent structure of 9, 10-dioxoanthracene. These chemical compounds possess strong laxative properties and occasionally show significant antioxidant effects, with the following variability order: Butylated hydroxyanisole (BHA) (96%) > anthrone (95%) > alizarin (93%) > aloe-emodin (78%) > rhein (71%) > emodin (36%) > anthraquinone (8%). Over 100 anthraquinone derivatives can be obtained from various *Cassia* species (19).

AGEs and RLAR bioassays were utilized to investigate the potent antidiabetic activity of anthraquinones obtained from the ethyl acetate fraction of *C. alata* seeds in vitro (19).

The water and ethanol extracts of *C. grandis* stems reduced the blood glucose levels to normal within 30 minutes of treatment, with values of 100.47 ± 1.36 and 108.39 ± 1.87 mg/dL, respectively, in the glucose tolerance test in rats at a dose of 150 mg/kg, compared to the standard antidiabetic agent glibenclamide, which reduced this parameter to 98.03 ± 0.02 mg/dL at 10 mg/kg (19).

*C. sieberiana* and *C. singueana* are utilized for treating diabetes, malaria, ulcers, and wound healing. Their extracts were evaluated for antioxidant effects through DPPH radical scavenging, FRAP, ABTS+ radical cation scavenging, β-carotene bleaching, and flavonoid and phenolic content assays. *C. sieberiana* exhibited significant activities for 15-LOX inhibition (80.93% inhibition) and FRAP (2120 μmol Fe2+/g dry extract). The extracts of *C. singueana* and *C.* *sieberiana* contain α-glucosidase inhibitors, antioxidants, and anti-inflammatory compounds, making them useful for the treatment of ulcers and diabetes (17).

Upon investigation of different fractions of *C. angustifolia* using an in-vitro AGI test with a concentration of 500 µg/mL, the hexane fraction and the remaining methanolic fractions demonstrated the highest inhibition. Additionally, the acarbose used in this measurement showed results similar to those of the hexane fraction and the remaining methanolic fractions, all reaching close to 50%. Considering the results of α-glucosidase enzyme inhibition, the hexane fraction of *C. angustifolia* can be a valuable component for further in-vitro and in-vivo anti-diabetic studies.

### 5.1. Conclusions

This preliminary study suggests that the hexane extract has a significant effect in inhibiting the α-glucosidase enzyme, but further phytochemical studies are needed to identify its active components as α-glucosidase inhibitors, which will be considered in future studies.

## Data Availability

The dataset presented in the study is available on request from the corresponding author during submission or after publication.
